# Determinants of Noncompletion of the Third Dose of Tetanus Toxoid Vaccine in Pregnant Women in Dschang Health District, Cameroon

**DOI:** 10.1155/2020/1603518

**Published:** 2020-02-25

**Authors:** Florent Ymele Fouelifack, Bruno Kenfack, Skinner Lekelem Nguefack, Jackson Jr Nforbewing Ndenkeh, Jeanne Hortence Fouedjio, Loic Dongmo Fouelifa, Pierre Marie Tebeu

**Affiliations:** ^1^Institute of Medical Technology of Nkolondom, Yaoundé, Cameroon; ^2^Obstetrics and Gynecology Unit of Yaoundé Central Hospital, Yaoundé, Cameroon; ^3^Research Education and Health Associative Group GARES-Falaise, Dschang, Cameroon; ^4^Faculty of Medicine and Pharmaceutical Sciences, University of Dschang, Dschang, Cameroon; ^5^Obstetrics and Gynecology Unit of District Hospital, Dschang, Cameroon; ^6^Center of International Health, Ludwig Maximilian University of Munich, Munich, Germany; ^7^Faculty of Medicine and Biomedical Sciences, University of Yaoundé I, Yaoundé, Cameroon; ^8^Faculty of Health Sciences, University of Lomé, Lomé, Togo; ^9^School of Armies Health Services of Lomé, Lomé, Togo; ^10^Centre Inter‐Etats d'Enseignement Supérieur en Sante Publique d'Afrique (CIESPA), Brazzaville, Congo

## Abstract

*Introduction*. Tetanus vaccination plays an important role in the fight against neonatal mortality. Our study aimed to determine the noncompletion rate of the 3^rd^ dose of tetanus toxoid vaccine (TTV) and to analyze the associated factors in pregnant women.

**Methods:**

This was a cross-sectional study conducted in 2 hospitals of Dschang Health District and targeting all women at least in their second gestation coming for antenatal consultation. Upon informed consent by the participant, a prepared questionnaire was administered. The collected data was analyzed using SPSS v22.0 with results presented in means and proportions. Logistic regression was used at two levels to identify independently associated factors for noncompletion of the third dose of TTV with a significance set at 5%.

**Results:**

A total of 380 pregnant women were recruited in this study of mean age 27 ± 5.2 yrs, 70% being married, more than 80% having at least secondary education, and 31.8% of them being students. It was noted that 172 (45.26%) of these women had not received the third dose of TTV. The analysis of the adjusted effects showed that not going to postnatal consultation (aOR = 6.75; 3.98–11.49, *p* < 0.0001), not accompanying her baby to vaccination (aOR = 3.784; 1.803–7.942, *p* < 0.0001), not accompanying her baby to vaccination (aOR = 3.784; 1.803–7.942, *p* < 0.0001), not accompanying her baby to vaccination (aOR = 3.784; 1.803–7.942,

**Conclusion:**

Tetanus vaccination coverage is not yet optimal in Dschang Health District and is associated with marital status as well as postgestational behavior of the mothers. There is thus the need to put in place strategies that will provide social support to single mothers as well as encourage women to attend postnatal consultation and to accompany their own children for vaccination. Furthermore, community-based vaccination could capture some of the lost women thus optimizing the overall vaccination coverage.

## 1. Introduction

Tetanus is a life-threatening disease that mostly affects low-income countries and populations with limited or no access to health services [1]. Every year around the world, tetanus is responsible for more than 450,000 child deaths in the first month of life and more than 40,000 mothers contract this infection during delivery [2]. Many authors found factors related to women, accessibility, organization of immunization services, and the technical skills of health care workers that influence the completion of tetanus vaccination in pregnant women [1, 3]. Implementation of tetanus prevention strategy based mainly on tetanus toxoid vaccination in pregnant women has reduced the number of deaths due to neonatal tetanus worldwide by 93%, from 787,000 cases in 1988 to 58,000 cases in 2010 [2]. Immunization coverage against tetanus remains low in many African countries, notably in Benin where the coverage rate in the second dose of tetanus toxoid vaccine (TTV) was 60% within the last five years, lower than the national objective of 86% [4].

In Cameroon, there is low coverage of TTV in pregnant women. According to the 4th Cameroon Demographic Health Survey back in 2011, 59% of women received at least two doses of TTV during their last pregnancy giving a 41% noncompletion rate of the 2^nd^ dose of TTV [5]. On the other hand, a study showed an incidence of a neonatal tetanus case for every 1000 live births within 2015 in the country [6]. With 118 cases of maternal or neonatal tetanus identified in 2015, it is worth noting that tetanus toxoid vaccination coverage that same year was 62% which was below optimal. The proposition of appropriate strategies to reduce TTV noncompletion rate would contribute to the elimination of maternal and neonatal tetanus in Cameroon [6] and therefore play a role in the achievement of the third sustainable development goal. In order to propose appropriate strategies to strengthen and improve the current vaccination coverage, there is need to understand the underlying factors of noncompletion. This study was thus implemented with the overall objective to appreciate the TTV coverage in Dschang Health District while determining the factors associated with noncompletion of the third dose of TTV.

## 2. Materials and Methods

### 2.1. Study Design, Time Period, and Place of Study

The study was a cross-sectional conducted in 2018 targeting pregnant women coming for antenatal consultation. Two hospitals were selected from Dschang Health District found in the West region of Cameroon, namely, the District Hospital of Dschang and “Saint Vincent de Paul” Hospital. Data collection in these hospitals lasted a month (the whole of February 2018). The above hospitals were chosen as the most frequented in the city of Dschang in terms of antenatal consultation and vaccination. They received daily about 15 and 12 women from different sociocultural backgrounds for antenatal consultation, respectively.

### 2.2. Sampling and Procedure

Sample size for this study was calculated using the following formula which gave 384:

(1) $$\begin{array}{c} n\;=\;\frac{Z^{2}\;p\;q}{\alpha^{2}}, \end{array}$$

where *Z* = Z-value which is 1.96, *p* = anticipated proportion of pregnant women who have not received the 3^rd^ dose of TTV in Cameroon (=50% if unknown), *q* = 1−*p*, and *α* = significance level set at 5%.

Notwithstanding, with the exhaustive sampling method within allocated time period for data collection, 380 pregnant women who were at least in their second gestation consented to participate in the study. All nulliparous women as well as women who did not consent to participate in the study were excluded. For each participant upon informed consent, a prepared questionnaire was administered which gathered information on their sociodemographic characteristics, knowledge on, and behavior related to tetanus prevention, etc. The dependent variable here was noncompletion of the third dose of TTV, and the tested independent variables included sociodemographic, economic, medical, and obstetrical characteristics of the participants.

### 2.3. Data Analysis

All the data collected were keyed in a data entry spreadsheet using Microsoft Excel 2010 software and analyzed using the software SPSS v22.0. The descriptive results were presented in means and proportions for continuous and categorical variables, respectively. Bivariate logistic regression was used to identify potential associated factors which were adjusted for each other's effect in a multivariate analysis with a significance set at 5%.

### 2.4. Ethical Considerations

Ethical clearance no. 1552 CEI-UDo/04/2018/T was obtained from the Ethical Committee of the University of Douala/Faculty of Medicine and Pharmaceutical Sciences. Administrative authorizations were also obtained from the directors of both hospitals prior to the start of study. Furthermore, each participant voluntarily consented before being included in the study while all information obtained was treated with respect of confidentiality. For minors, in addition to parent consent, it was required they too provided their assent before being included in the study.

## 3. Results

In total, 380 participants were included in this study with 188 (49.47%) and 192 (50.52%) recruited from Dschang District Hospital and St. Vincent Hospital, respectively. This study population had age ranging from 17 to 40 yrs with mean age being 27 ± 5.2 yrs and majority fell in the age group 20–35 yrs (Table 1). More than half of the women were workers in public or private sectors, more than three quarters were at least having secondary education, and more than two thirds were married. Overall, it was noted that 17 (4.5%) of the participants had never received tetanus vaccine before. Furthermore, it is worth noting that only 208 (54.74%) had received the third dose, giving a TTV noncompletion rate of 45.26%.

### 3.1. Sociodemographic and Obstetrical Characteristics of Participants according to the Number of Doses of TTV

As shown in Table 1, health facility (0,014), marital status (0,002), parity (<0.0001), number of antenatal consultations during the last pregnancy (<0.0001), and health facility where ANC was attended (<0.0001) were all significantly associated with the noncompletion of the 3rd dose of TTV. There was no statistically significant association between the noncompletion of 3rd dose of TTV and profession as well as level of education (*p* values of 0.278 and 0.676, respectively).

### 3.2. Knowledge of Participants on Tetanus according to the Number of TTV

As shown in Table 2, awareness on the existence of tetanus, knowledge of the recommended dose for protection, being informed on the necessity to receive a third dose of TTV during their antenatal consultations, attendance of postnatal consultation, and being the person accompanying baby for vaccination were all significantly associated with the noncompletion of the third dose of TTV (*p* value of <0.0001). Awareness of the disease on the other hand was not significantly associated with the noncompletion of the third dose of TTV (*p* value of 0.271).

### 3.3. Adjusted Effects of Factors Influencing the Number of Doses of TTV

When all significant factors above were adjusted for each other's effect, it was noted that three of the factors remained independently associated with the noncompletion of the third dose of TTV (Table 3). Nonattendance of postnatal consultation (aOR: 6.75 CI = 3.98–11.49, *p* < 0,0001), being single (aOR: 1.87 CI = 1.05–3.3, *p*=0.034), and not being the person who accompanies the baby for vaccination (aOR: 3.85 CI = 1.84–8.05, *p* < 0.0001) were all significantly associated with not completing the third dose of TTV. Therefore, compared to married women, single women were almost twofold not liable to receive the third dose of TTV. Women who did not attend postnatal consultation were 6.7 times more liable of not receiving the third dose of TTV compared to women who attended postnatal consultation. Furthermore, as compared to women who accompanied their babies for vaccination, women who did not accompany their babies for vaccination were almost four times more liable not to receive the third dose of TTV.

## 4. Discussion

Neonatal tetanus remains a public health problem in developing countries despite being a disease evitable by vaccination. This challenge in the elimination of tetanus transmission from mother to child is due to noncompletion of vaccination. This study was thus implemented to determine the noncompletion rate of TTV as well as identify independently associated factors in the context of Dschang Health District.

### 4.1. Noncompletion Rate of the 3rd Dose of TTV

Women who were never vaccinated accounted for 4.5% in our sample. This result is lower than that of 6.1% found in Benin by Togora et al. in 2013 [4]. His study however was carried out in different context and concerned mainly the completion of the second dose of TTV. It could also be possible that with time women became more aware and were advised to start tetanus vaccination which thus could justify the drop in percentage of those never vaccinated for tetanus. It was also noted that 172 (45.26%) had received less than three doses of TTV. In the literature, we did not find any studies on the noncompletion of the third dose of TTV to compare with our results. However, several authors have studied the completion of the second dose of TTV. Our rate of 45.26% noncompletion in the third dose of TTV is lower than those found for the noncompletion of the second dose of TTV which were 66.7% by Hamadoun et al. in Mali in 2006 [7], 61.7% by Togora et al. in Benin in 2013 [4, 8], and 49.3% by Talanie et al. in Congo in 2015 [9]. We believe that the above noncompletion rate is pretty high thus justifying the need to educate women about the importance of getting the third dose of TTV after delivery.

### 4.2. Socioeconomic and Obstetrical Characteristics of Pregnant Women Who Have Not Received the Third Dose of TTV

There was no difference between age groups in our study (Table 1). This is different from the result found by Hamadoun et al. where the 14–23 yrs age group was the most represented. This difference can be explained by the fact that we excluded nulliparous women in our study contrary to their study where all women in childbearing age were included [7, 8].

Married women were 2.43 times more likely to have received the 3rd dose of TTV and more than those who were single. This could be due to the material and psychosocial support given to the married woman. This result is close to that of Togora et al. who found in 2013 that married women were 5.33 times more likely to have good coverage in second dose of TTV and more than those who are single. This result justifies the need to encourage men to support spouses in their health and to encourage unmarried women to meet their vaccination schedule.

All women had at least one antenatal consultation during their last pregnancy. This is different from the result obtained by Hamadoun et al., who found in 2006 in Mali that the number of women who did not have antenatal consultation was 10.3% [7]. This rate of attendance of antenatal consultation could be explained by the fact that our study was conducted in an urban area where women are better informed and homes are usually close to the hospital. This result obtained by our study is a proof of the effectiveness of antenatal consultation services in our health facilities.

We found that 48.7% of women did not know the maximum dose of TTV needed to be protected. Hamadoun et al. found 61.8% [7]. This difference could be explained by the communication which may be better in our context [7]. It is therefore important to emphasize the awareness of this disease and the means of prevention.

In our study, the level of instruction had no effect on vaccination status. In contrary, Hamadoun et al. [7] showed that the more the women are educated, the better they are vaccinated. The same observation was made in the 2010–11 Senegal Demographic and Health and Multiple Cluster Indicator [10] where the birth protection level of mothers of medium or higher level was 68% compared to 62% of mothers not attending school.

### 4.3. Factors Influencing the Noncompletion of the 3rd Dose of Tetanus Toxoid Vaccine in Pregnant Women

The first factor associated with noncompletion of tetanus vaccine was “not going to postnatal consultation” (aOR = 6.75 CI: 3.98–11.49, *p* < 0.0001). This could be due to the fact that the 3^rd^ dose of TTV that is done six months after the second (usually after giving birth) is not respected by these women as they think the vaccine is made for the pregnant woman only. By going to a postnatal consultation, they are exposed to the caregivers who explain to them and remind them of the need to get vaccinated. We did not find any studies in the literature with an association between postnatal consultation and TTV.

The fact of not always accompanying their baby in vaccination was also associated with noncompletion of the 3rd dose of TTV (aOR = 3784 IC: 1.803–7.942, *p* < 0.0001). This can be explained by the fact that, during the vaccination of the baby, the women are more educated and reminded about the necessity of the vaccine. Women who forgot their TTV appointment or who thought that the TTV was only for the pregnant women can be reminded and subsequently be vaccinated.

The above two factors justify the need to educate women on the importance of going for postnatal consultation as well as accompanying their own children for vaccination thus giving caregivers the opportunity to remind them of their own tetanus vaccination appointment and subsequently following them up to ensure they respect it.

Contrary to our results, authors have not found an association between accompanying the baby in vaccination and completion of the third dose of TTV. This is the case of Togora et al. who rather found an association between the number of antenatal consultations, the knowledge of the vaccination schedule, the use of radio and television at least once a week, marital status, profession, fear of side reactions (no/yes), and explanation of the vaccination schedule by the provider to be associated with coverage of the second dose of TTV [4, 7, 8, 11].

Lastly our study found an association between noncompletion of the third dose of TTV and marital status where single mothers had a higher chance of not completing the third dose of TTV. A possible explanation for this could be that single mothers especially those who had undesired pregnancies will not have the social support as well as the financial means and thus would find it difficult to go back to hospital for postnatal consultation in general and the third dose TTV in particular. It is also worth noting that factors like place of antenatal consultations, number of antenatal consultations during the last pregnancy, being informed about the third dose of TTV during the antenatal consultation, and the appointment for the third dose of TTV on the notebook were identified with the bivariate logistic regression analysis but when adjusted for all other identified factors, they were no more statistically significant. All the above independent and/or potential factors were identified by Kalac and Yalc [13], Painvin et al., Talani et al. [9] to be associated with completion of the second dose of TTV.

### 4.4. Limitations of the Study

The study was conducted in two health facilities, all in the urban area of Dschang Health District, which could lead to selection bias. Notwithstanding, the selected health facilities receive patients even from the hinterlands and from a wide cultural backgrounds which somehow gives second degree image on the reality of the situation in the health district. Also some questions required the participant to recall previous knowledge, thus the possibility of memory bias which nonetheless was minimised by acquiring some of the long term information from their hospital books if available and not insisting on an answer if participant did not remember.

## 5. Conclusion

The noncompletion rate of the third dose of tetanus vaccine was 45.26% which is pretty high especially if there is need to attain the optimal vaccination coverage to greatly reduce maternal and neonatal tetanus. The factors associated with the above noncompletion rate were being single, nonattendance of postnatal consultation, and not accompanying the baby for vaccination. There is thus the need to put in place strategies that will provide social support to single mothers making them able to not only look after their babies but themselves too as well as encourage women to attend postnatal consultation and to accompany their own children for vaccination, these later providing the opportunity for caregivers to inform, educate, and communicate to them about the necessity for them to respect appointment for their 3^rd^ dose of TTV. Furthermore, tetanus vaccination could be integrated in community-based activities like outreaches so as to capture some of the women identified to have been lost to tetanus vaccination follow-up thus optimizing the overall vaccination coverage.

## Figures and Tables

**Table 1:** Sociodemographic and obstetrical characteristics of participants according to the number of doses of TTV.

Variables	Number of doses of TTV						OR (95% CI)	*p* value
	<three doses		≥three doses		Total			
	*N* = 172		*N* = 208		*N* = 380			
	*n*	%	*n*	%	*n*	%		
*Age* (*mean* *±* *SD*)	26.17 ± 4.85 yrs		28.79 ± 5.26 yrs		27 ± 5.2 yrs			

*Age groups*								0.005
17–19	8	4.7	5	2.3	13	3.4	3.07 (0.78–11.49)	
20–25	81	47.1	65	31.3	146	38.4	2.28 (1.11–5.15)	
26–35	71	41.2	115	55.3	186	48.9	1.98 (0.62–6.75)	
35 and above	12	7	23	11.1	35	9.3	1	

*Health facilities*								0,014
District hospital	97	51.6	91	48.4	188	49.47	1.66 (1.11–2.49)	
St. Vincent hospital	75	39.06	117	60.94	192	50.52	1	

*Profession*								0.278
Housewife	18	10.5	25	12.0	43	11.3	1.49 (0.64–3.47)	
Student	62	36.0	59	28.4	121	31.8	2.17 (1.08–4.34)	
Private/public salary earner	92	53.5	124	59.6	216	56.8	1	

*Level of education*								0.676
Primary	29	16.8	30	14.4	59	15.5	1.25 (0.71–2.46)	
Secondary	93	54.1	110	52.9	203	53.4	1.14 (0.72–1.81)	
Higher	50	29.1	68	32.7	118	31.1	1	

*Marital status*								0,002
Single	69	40.1	45	21.6	114	30.0	2.43 (1.54–3.84)	
Married	103	59.9	163	78.4	266	70.0	1	

*Parity*								<0.0001
Paucipara	94	54.6	71	34.1	165	43.4	2.2 (1.11–3.36)	
Multipara	66	38.4	117	56.3	183	48.2	1.23 (0.78–2.23)	
Grand multipara	12	7	20	9.6	32	8.4	1	

*Number of antenatal consultations during the last pregnancy*								<0.0001
≤2	35	79.54	9	20.45	44	11.57	5,64 (2,63–12,12)	
≥3	137	40.77	199	59.22	336	88.42	1	

*Health facility where antenatal consultation was done*								<0.0001
Integrated health center	11	68.75	5	31.25	16	4.21	2.48 (0.82–7.69)	
Private health center	25	71.42	10	28.57	35	9.21	2.82 (1.27–6.25)	
District hospital	77	47.23	86	52.77	163	42.89	1.15 (0.76–1.7)	
Confessional hospital	59	35.75	106	64.24	168	43.42	1	

Paucipara: 1-2 deliveries; multipara: 3–5 deliveries; grand multipara: more than 5 deliveries; ANC: antenatal consultations.

**Table 2:** Knowledge of participants on tetanus according to the number of doses of TTV.

Variables	Number of doses of TTV						OR (95% CI)	*p* value
	<three doses		≥three doses		Total			
	*N* = 172		*N* = 208		*N* = 380			
	*n*	%	*n*	%	*n*	%		
*Have you ever heard of tetanus*?								0.271
No	1	0.58	0	0	1	0.3	—	
Yes	171	99.42	208	100	379	99.7	1	

*Awareness of the existence of TTV*								<0.0001
No	14	8.14	0	0	14	3.7	3.37 (1.88–5.69)	
Yes	158	91.86	208	100	366	96.3	1	

*Vaccinated against tetanus*?								<0.0001
No	17	9.88	0	0	17	4.5	—	
Yes	155	99 .11	208	100	363	95.5	1	

*Knowledge of the recommended doses for definite protections*								<0.0001
No	114	66.28	70	33.65	184	48.7	3,87(2.52–5.94)	
Yes	57	33.72	137	66.34	194	51.3	1	

*Information on TT3 during antenatal consultations*								<0.0001
No	124	58.18	186	41.87	310	81.57	3.37 (1.88–5.69)	
Yes	50	29.44	20	30.55	70	18.43	1	

*Postnatal consultation*								<0.0001
No	132	70.96	54	29.03	186	48.94	9.43 (5.37–15.0)	
Yes	40	20.61	154	79.38	194	51.05	1	

*Who accompanies the baby for vaccination*?								<0.0001
Someone else	120	69,8	194	93,3	314	82.6	17.76 (6.9–645.7)	
me	52	30,2	14	6,7	66	17.4	1	

**Table 3:** Adjusted effects of factors influencing the number of doses of TTV.

Variables	Number of doses of TTV				aOR (95% CI)	*p* value
	<three doses		≥three doses			
	*N* = 172		*N* = 208			
	*n*	%	*n*	%		
*Health facilities*						0.296
District hospital	97	51.6	91	48.4	0.58 (0.223–1.5)	
St. Vincent hospital	75	39.06	117	60.94	1	

*Age group*						0.441
≤19	8	61.5	5	38.4	1.62 (0.12–8.3)	
20–25	81	55.48	65	44.5	1.26 (0.46–3.48)	
26–35	71	38.17	115	61.83	0.79 (0.296–2.2)	
≥36	12	34.28	23	65.7	1	

*Marital status*						**0.034**
Single	70	61.4	44	38.6	1.87 (1.05–3.3)	
Married	103	38.7	164	61.3	1	

*Postnatal consultation*						**<0.0001**
No	132	70.96	54	29.03	6.75 (3.98–11.49)	
Yes	40	20.61	154	79.38	1	

*Health facility where antenatal consultations were done*						0.088
Integrated health center	11	68.75	5	31.25	3.33 (0.7–14.28)	
Private health center	25	71.42	10	28.57	1.25 (0.41–3.75)	
District hospital	77	47.23	86	52.77	3,43 (1.23–9.51)	
Confessional hospital	59	35.75	106	64.24	1	

*Number of antenatal consultations during the last pregnancy*						0.116
≤2	35	79.54	9	20.45	2.17 (0.88–0.57)	
≥3	137	40.77	199	59.22	1	

*Information on TT3 during antenatal consultations*						0.376
No	124	58.18	186	41.87	1.43 (0.64–3.18)	
Yes	50	29.44	22	30.55	1	

*RDV for TT3 written on the hospital booklet*						0.101
I do not know	23	71.87	9	28.12	2.72 (0.73–7.8)	
No	31	75.6	10	24.39	2.09 (0.73–5.4)	
Yes	118	38.4	189	61.5	1	

*Who accompanies the baby for vaccination*?						**<0.0001**
Someone else	120	69.8	194	93.3	3.85 (1.84–8.049)	
Myself	52	30.2	14	6.7	1	

## Data Availability

The data used to support the findings of this study are available from the corresponding author upon request.
